# scAGCI: an anchor graph-based method for cell clustering from integrated scRNA-seq and scATAC-seq data

**DOI:** 10.1093/bib/bbaf244

**Published:** 2025-07-07

**Authors:** Yao Dong, Jiaxue Zhang, Jin Shi, Yushan Hu, Xiaowen Cao, Yongfeng Dong, Xuekui Zhang

**Affiliations:** School of Artificial Intelligence, Hebei University of Technology, No. 5340, Xiping Road, Beichen District, Tianjin 300401, China; Hebei Engineering Research Center of Data-Driven Industrial Intelligent, No. 5340, Xiping Road, Beichen District, Tianjin 300401, China; Hebei Province Key Laboratory of Big Data Computing, No. 5340, Xiping Road, Beichen District, Tianjin 300401, China; School of Artificial Intelligence, Hebei University of Technology, No. 5340, Xiping Road, Beichen District, Tianjin 300401, China; School of Artificial Intelligence, Hebei University of Technology, No. 5340, Xiping Road, Beichen District, Tianjin 300401, China; Department of Mathematics and Statistics, University of Victoria, 3800 Finnerty Road PO Box 1700 STN CSC, Victoria BC V8W 3P2, Canada; School of Artificial Intelligence, Hebei University of Technology, No. 5340, Xiping Road, Beichen District, Tianjin 300401, China; School of Artificial Intelligence, Hebei University of Technology, No. 5340, Xiping Road, Beichen District, Tianjin 300401, China; Hebei Engineering Research Center of Data-Driven Industrial Intelligent, No. 5340, Xiping Road, Beichen District, Tianjin 300401, China; Hebei Province Key Laboratory of Big Data Computing, No. 5340, Xiping Road, Beichen District, Tianjin 300401, China; Department of Mathematics and Statistics, University of Victoria, 3800 Finnerty Road PO Box 1700 STN CSC, Victoria BC V8W 3P2, Canada

**Keywords:** cell clustering, single-cell, dynamic anchor graph, scRNA-seq, scATAC-seq

## Abstract

Single-cell multi-omics clustering confronts noise and heterogeneity barriers. Current multi-view anchor graph approaches, though successful in noise reduction, inadequately model higher order feature interactions. To address this issue, we propose scAGCI, a cell clustering method based on anchor graphs that integrates both scRNA-seq and scATAC-seq data. Our method captures specific and shared anchor graphs representing the properties of omics data in the process of dynamic anchor unification, and mines high-order shared information to complete the omics representation. Subsequently, clustering results are obtained by integrating the specific and shared omics representation. Benchmarking against 13 state-of-the-art methods confirms scAGCI’s superior clustering performance and computational efficiency in cell-type identification and subtype resolution. The method preserves biologically meaningful omics patterns, as evidenced by marker gene enrichment and functional analyses, establishing it as a robust tool for elucidating cellular heterogeneity in single-cell multi-omics data.

## Introduction

Single-cell multi-omics clustering is an important part of single-cell analysis, as it typically integrates the characteristics of different omics data for cluster analysis. For instance, the characteristics of transcriptome data and proteome data are used to cluster cells together [1], providing a more comprehensive understanding of cell function and characteristics. In addition, single-cell multi-omics cell clustering can facilitate the discovery of correlations and complementarities between different omics data, thereby enhancing our insight into the regulatory mechanisms governing various biological processes within cells.

Heterogeneity and high noise pose significant challenges in single-cell multi-omics clustering. Each omic has its biases, e.g. scRNA-seq data are affected by environmental RNA and dropout issues, while epigenomic data (such as scATAC-seq) involve sparse binary or nearly binary measurements [2, 3]. Additionally, the number of features measured varies widely, ranging from tens or hundreds in spatial and protein data to tens of thousands in scRNA-seq data [4, 5].

To address these issues, researchers have devoted considerable efforts. The approaches can be categorized into four main computational paradigms: matrix decomposition and factor analysis-based methods, encoder-based methods, contrastive learning-based methods, and graph-based methods.

The methods based on matrix decomposition and factor analysis including SLNMF [6], MOFA+ [7], scAI [8], Seurat v4 [9], scABC [10], Liger [11], and UnionCom [12] directly obtain the shallow low-dimensional information shared among the omics, but fail to attain the nonlinear relationship between the omics [13]. Therefore, the encoder methods such as DCCA [14], scMVAE [15], scMVP [16], and scMoMtF [17] are introduced to explore the nonlinear relationship between omics. These methods work by building different encoders, such as zero dilatation negative binomial ZINB encoders [18], VAE with probabilistic Gaussian mixture models [19, 20], etc. They iteratively learn the underlying representation in the data to reveal the distribution of different omics further, and then reconstruct the data through the decoder to maximize information retention and reduce noise. Further, the researchers implement contrastive learning [21, 22] between encoders to extract shared information from scRNA-seq and scATAC data for fusion. Single-cell multi-omics data exhibit complex associations and interactions. While these encoders can reduce dimensionality to a low-dimensional space, they also result in the loss of relationships between the data. Graph learning like scMoGNN [23], GLUE [24], MarsGT [25], MST-GAT [26], and SIMBA [27] is introduced for clustering single-cell multi-omics data, enabling the capture of relationships, including similarity and connectivity between cells, to more accurately describe internal structure. However, existing methods still lack mining and utilization of shared information from multi-omics data, indicating that further refinement is needed in mining omics information.

With the continuous advancement of sequencing technology, another major challenge of single-cell multi-omics is the expanding scale of data. As graph networks aggregate rich information, their computing costs are increasing exponentially. Existing clustering methods suffer from insufficient utilization of graph information and high network computing complexity, leading to increased consumption of computing resources and limitations in clustering performance.

To address all of the above issues, we develop scAGCI, an anchor graph-based method for cell clustering from integrated scRNA-seq and scATAC-seq data. Inspired by anchor learning in the multi-view domain, we propose a strategy to collaboratively optimize anchor learning and graph representation for the fused representation of single-cell multi-omics. scAGCI effectively extracts specific and mine high-order information shared information from multiple omics sources to represent scRNA-seq and scATAC-seq data and organically integrates this information to obtain clustering results.

## Related work

Multi-omics can be processed as a way of multi-view. In the field of multi-view analysis, it has been observed that existing multi-view clustering algorithms are primarily based on graph models, which are time-consuming and challenging to apply to large-scale datasets in practice [28]. To enhance efficiency, researchers have proposed utilizing the relationship between anchor and data to represent the relationships among all data.

Currently, there are two types of anchor graph learning: heuristic sampling strategy [29, 30] and adaptive sampling strategy [31, 32]. Heuristic sampling strategies have been employed in the realm of single-cell omics, where researchers have introduced a scalable and efficient anchor graph clustering algorithm [33] for scRNA-seq data. The algorithm enhances the runtime and scalability of state-of-the-art (SOTA) consensus clustering methods while maintaining accuracy. However, this strategy often relies on K-means or random sampling, leading to clustering results that depend on initialization and may not represent the data.

We pay more attention to the adaptive anchor selection strategy. EOMSC-CA [32] applied efficient multi-view subspace clustering and consensus anchor learning. The researchers unify the anchor selection process and the graph construction process for joint optimization, obtaining a representative consensus anchor graph. In the field of multi-omics, this approach is obviously not applicable. Firstly, in the learning process, this method uses the graph structure information. While in the field of single cells, features contain rich biological information that mainly distinguishes cell types. Secondly, the method learns a shared representation from different views, and completely ignores the differences between different views. In the field of multi-omics, the feature expression of cells varies greatly at different molecular levels. The shared representation not only helps us to get the connection across the omics but also misses the differences of the omic graph. Supplementary Figure S1 illustrates the differences between the EOMSC-CA and our proposed method.

## Methods

**Figure 1:**
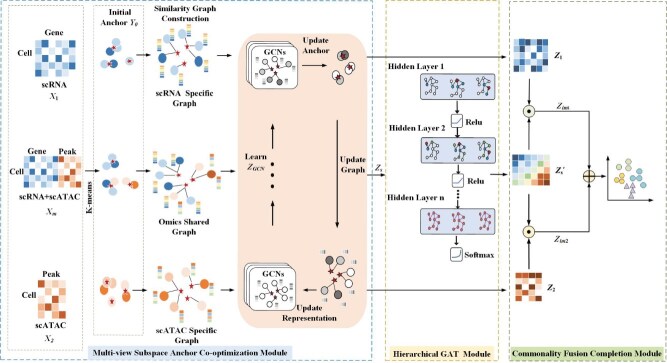
The architecture of scAGCI.

We develop scAGCI, an anchor graph-based method for cell clustering from integrated scRNA-seq and scATAC-seq data. It consists of three parts: multi-view subspace anchor co-optimization module, hierarchical GAT module, and commonality fusion completion module. The architecture is shown in Fig. 1.

### Multi-view subspace anchor co-optimization module

#### Specific anchor graph construction

With the single-cell data, the specific anchor graph is constructed in each omic. In our study, we regard scRNA-seq data as $X_{1}$, scATAC-seq as $X_{2}$, and their merge data as $X_{m}$ to form views, where $X_{i} \in \mathbb{R}^{d_{i} \times n},i=1,2$ represents the $i$th view data with $d_{i}$ dimensions and $n$ cells.

Assume that a linear combination of other data points in the same subspace can express each data point. The subspace clustering is to construct a graph


(1) \begin{align*}& \min_{Z} L(X, XZ) + \rho \phi(Z),\end{align*}

where $L(\cdot )$ and $\phi (\cdot )$ represent the loss function and the regularization function, respectively, and $\rho $ controls the trade-off between the two terms. $Z\in \mathbb{R}^{n \times n}$ denotes the representation matrix, encoding the relationships between data points onto subspaces.

Define the specific anchor graph as $B_{i}(X_{i}, Y_{i}, Z_{i}),i=1,2$ using bipartite graph. As an extension of subspace clustering, the bipartite graph anchor subspace [32] clustering can be expressed as


(2) \begin{align*}& \min_{Z_{i}} L(X_{i}, Y_{i} Z_{i}) + \rho \phi(Z_{i}),\end{align*}

where $Y_{i}\in \mathbb{R}^{d_{i} \times m},i=1,2$ denotes the specific anchor matrix on $X_{1}$ and $X_{2}$. We start a subset of $m$ clusters from all $n$ cells as initial anchors $Y_{0}$, which are the centroids of the identified clusters by applying k-means [34] to the above random subsample. $Z_{i}\in \mathbb{R}^{m \times n}$ is the specific anchor graph representation, reflecting the relationship between $Y_{i}$ and $X_{i}$.

The Frobenius norm is used as the loss function. The above bipartite graph anchor subspace clustering is conducted as follows:


(3) \begin{align*} \min_{Z_{i}} \|X_{i} - Y_{i} Z_{i} \|_{F}^{2} + \rho \|Z_{i}\|_{F}^{2} ; \nonumber\\ \quad \text{s.t.} \quad Z_{i} \geq 0,\quad{Z_{i}}^{\mathrm{T}} \mathbf{1} = \mathbf{1},\end{align*}

where $\mathbf{1}$ is a column vector of ones, ensuring that the sum of each column is 1. The omic-specific anchor graph $B_{i}$ is obtained.

#### Shared anchor graph construction

To integrate omics information and obtain shared views, we define the anchor projection matrix $W_{i}\in \mathbb{R}^{d_{i} \times d}$ to align shared anchor $Y_{s}$ with the original data of the $i$th omics. The shared graph is established among multiple omics data:


(4) \begin{align*} &\begin{split} &\min_{\boldsymbol{\alpha},W_{i},Y_{s},Z_{s}} \sum_{i=1}^{v} \alpha_{i}^{2} \left\|X_{i} - W_{i} Y_{s}Z_{s}\right\|_{F}^{2} + \|Z_{s}\|_{F}^{2},\ \quad\\ &\quad\text{s.t.} \quad \boldsymbol{\alpha}^{\mathrm{T}} \mathbf{1} = 1,\quad W_{i}^{\mathrm{T}} W_{i} = I_{d},\quad Y_{s}^{\mathrm{T}} Y_{s} = I_{m},\\ &\quad \quad \quad Z_{s} \geq 0, \quad Z_{s}^{\mathrm{T}} \mathbf{1} = \mathbf{1}, \end{split}\end{align*}

where $v$ denotes the number of views, $\boldsymbol{\alpha }=\{\alpha _{1},...,\alpha _{v}\}^{T}$  $\alpha _{i}$ is to balance the influence between omics. $Z_{s}$ is the shared anchor graph representation, reflecting the relationship between the anchor sets and omics.

#### Anchor selection and graph representation cooperative optimization

To improve anchor graph-based multi-view subspace methods, we propose a cooperative optimization of graph representation and anchor selection. This approach aims to eliminate the disconnect between them and reduce uncertainty in the clustering structure. It can learn both shared and specific features of omics information while ensuring consistency in the anchors within high-dimensional data.

In the optimization process, we learn each view graph representation $Z_{GCN}^{i}$ using widely employed GCNs:


(5) \begin{align*}& Z_{GCN}^{i} = \sigma\left({D_{i}}^{\prime -\frac{1}{2}} {A}_{i}^{\prime} {D_{i}}^{\prime -\frac{1}{2}}H_{i}^{(l-1)} {W_{i}}^{\prime (l)}\right),\end{align*}

where $H_{i}^{(l-1)}$ is the node representation matrix (feature matrix) of $i$th view data at layer $l-1$, and ${W_{i}}^{\prime (l)}$ is the weight matrix at layer $l$. $A_{i}^{\prime} = A_{i} + I$ is the adjacency matrix $A_{i}$ of $i$th view data augmented with self-connections, forming a diagonal matrix. $D_{i}^{\prime}$ is the degree matrix of $A_{i}^{\prime}$. $\sigma $ is the activation function, typically Relu.


(6) \begin{align*}& Z_{s}= \sum_{i=1}^{v} \alpha_{i} Y_{s}^{\mathrm{T}} {Z_{GCN}^{i}}^{\mathrm{T}}.\end{align*}

With $Z_{s}$, $Y_{s}$, and $\alpha _{i}$ being fixed, $W_{i}$ can be optimized as


(7) \begin{align*} \min_{W_{i}} \sum_{i=1}^{v} \alpha_{i}^{2} \left\|X_{i} - W_{i} Y_{s}Z_{s}\right\|_{F}^{2} \quad \text{s.t.} \quad W_{i}^{\mathrm{T}} W_{i} = I_{d};\end{align*}

as $W_{i}$ is independent in each view, we can express Equation (7) in the following equivalent formulation:


(8) \begin{align*} \max_{W_{i}} \operatorname{Tr}\left[W_{i}^{\mathrm{T}} X_{i} Z_{s}^{\mathrm{T}} Y_{s}^{\mathrm{T}}\right] \quad \text{s.t.} \quad W_{i}^{\mathrm{T}} W_{i} = I_{d},\end{align*}

where $\mathrm{Tr}(\cdot )$ represents the trace of a matrix.

With $W_{i}$, $Y_{s}$, and $\alpha _{i}$ being fixed, $Z_{s}$ can be optimized as


(9) \begin{align*} \quad \min_{Z_{s}} \sum_{i=1}^{v} \alpha_{i}^{2} \text{Tr} [Z_{s}^{\mathrm{T}} Z_{s} - 2X_{i}^{\mathrm{T}} W_{i} Y_{s}Z_{s}], \nonumber\\ \text{s.t.} \quad Z_{s}\geq 0, \quad Z_{s}^{\mathrm{T}} \mathbf{1} = \mathbf{1}.\end{align*}

Similarly, with $Z_{s}$, $W_{i}$, and $\alpha _{i}$ being fixed, $Y_{s}$ can be expressed as


(10) \begin{align*} \max_{Y_{s}} \text{Tr}\left[Y_{s}^{\mathrm{T}} \sum_{i=1}^{v} \alpha_{i}^{2} W_{i}^{\mathrm{T}} X_{i} Z_{s}^{\mathrm{T}}\right] \quad \text{s.t.} \quad Y_{s}^{\mathrm{T}} Y_{s} = I_{m}.\end{align*}

When $Z_{s}$, $W_{i}$, and $Y_{s}$ are fixed, the objection function with respect to $\alpha _{i}$ can be formulated as follows:


(11) \begin{align*} \min_{\boldsymbol{\alpha}} \sum_{i=1}^{v} \alpha_{i}^{2} \left\|X_{i} - W_{i} Y_{s}Z_{s}\right\|_{F}^{2}, \nonumber \\ \quad \text{s.t.} \quad \boldsymbol{\alpha}^{\mathrm{T}} \mathbf{1} = 1, \quad \alpha_{i} \geq 0.\end{align*}

Define $O_{i} = \left \|X_{i} - W_{i} Y_{s}Z_{s}\right \|_{F}^{2}$; the optimal $\alpha _{i}$ can be obtained using the Cauchy–Buniakowsky–Schwarz [35] inequality, given by


(12) \begin{align*}& \alpha_{i} = \frac{1/O_{i}}{\sum_{i=1}^{v} \frac{1}{O_{i}}}.\end{align*}

Using the same method, optimizing Equation (3) allows us to obtain the representation of the specific graph representation $Z_{1}$ and $Z_{2}$.

### Hierarchical GAT module

Current research highlights the importance of prior knowledge in single-cell multi-omics, such as gene–gene co-regulation and peak interactions, to construct graph networks. In contrast, our method extracts shared higher order genomic information ($Z_{s}^{\prime}$) using multi-layer graph attention networks (GATs) to capture high-order gene interaction information. Specifically, the Hierarchical GAT [26] module employs a masked self-attention mechanism.

The input $Z_{s}$ to the graph attention layer is the node feature set $H=\{h_{1},h_{2},\ldots ,h_{n}\}$, where $ h_{i} \in \mathbb{R}^{F}$, and the output is $ H^{\prime k}=\{h_{1}^{\prime k},h_{2}^{\prime k},\ldots ,h_{n}^{\prime k}\}$, where $h_{j}^{\prime k} \in \mathbb{R}^{F^{\prime}}$, $n $ is the number of cells, $h_{i}, h_{j}^{\prime k} $ are the input and output node embedding of $k$th layer, and $ F, F^{\prime} $ are the dimensions of input and output node embedding. The normalized input and output node embedding can be represented as


(13) \begin{align*} & \overrightarrow{H} = \{\overrightarrow{h}_{1},\overrightarrow{h}_{2},\ldots,\overrightarrow{h}_{n}\}, \overrightarrow{h}_{i} \in \mathbb{R}^{F}; \end{align*}

(14) \begin{align*} & \overrightarrow{H}^{\prime} = \{\overrightarrow{h}_{1}^{\prime k},\overrightarrow{h}_{2}^{\prime k},\ldots,\overrightarrow{h}_{n}^{\prime k}\}, \overrightarrow{h}_{j}^{\prime k} \in \mathbb{R}^{F^{\prime}}. \end{align*}

To calculate the weight of each neighboring node, a shared weight matrix $\mathbf{W}^{k}$ is applied to each node through an attention mechanism at $k$th layer. The attention coefficient is calculated as follows:


(15) \begin{align*}& e_{ij}^{k} = a(\mathbf{W}^{k} \overrightarrow{h}_{i}^{k-1}, \mathbf{W}^{k}\overrightarrow{h}_{j}^{k-1}),\end{align*}

where $a$ represents the importance of node $j$ relative to node $i$. Normalizing the weight coefficients


(16) \begin{align*}& a_{ij}^{k} = \text{softmax}(e_{ij}^{k}) = \frac{\exp(e_{ij}^{k})}{\sum_{l \in n_{i}} (e_{il}^{k})},\end{align*}

applies the LeakyReLU, then


(17) \begin{align*}& a_{ij}^{k}= \frac{\exp\left(\text{LeakyReLU}\left(\overrightarrow{a}^{T} \left[\mathbf{W}^{k} \overrightarrow{h}_{i}^{k-1} || \mathbf{W}^{k}\overrightarrow{h}_{j}^{k-1}\right] \right)\right)} {\displaystyle{\sum_{l \in n_{i}} \exp\left(\text{LeakyReLU}\left(\overrightarrow{a}^{T} \left[\mathbf{W}^{k} \overrightarrow{h}_{i}^{k-1} || \mathbf{W}^{k} \overrightarrow{h}_{l}^{k-1}\right] \right)\right)}},\end{align*}

where $\overrightarrow{a}$ is the normalized attention coefficient calculated by $a$, $n_{i}$ is the neighborhood of nodes in the graph, and || represents concatenation. The representation of node $i$ is then given by


(18) \begin{align*}& \overrightarrow{h}_{i}^{\prime k} = \sigma\left(\sum_{j \in n_{i}} \overrightarrow{a}_{ij}^{k}\mathbf{W}^{k} \overrightarrow{h}_{j}^{k-1}\right) .\end{align*}

Using a multi-head attention mechanism, the final representation is obtained by taking the average


(19) \begin{align*}& \overrightarrow{h}_{i}^{\prime (k,q)} = \sigma\left(\frac{1}{Q} \sum_{q=1}^{Q} \sum_{j \in n_{i}} \overrightarrow{a}_{ij}^{k} \mathbf{W}^{k} \overrightarrow{h}_{j}^{k-1}\right),\end{align*}

where $Q$ is the number of attention heads; the hierarchical GAT module updates the hidden states of all nodes in the shared graph, allows the network to learn high-order neighboring node information, captures hierarchical graph node information, and obtains high-order shared graph representation $Z_{s}^{\prime}$.

### Commonality fusion completion module

We design a commonality fusion completion operator to integrate information from the specific anchor graph representation $Z_{1}$ and $Z_{2}$ and the high-order shared graph representation $Z_{s}^{\prime}$, further enriching the representation of the specific graph:


(20) \begin{align*} & Z_{im1} = Z_{s}^{\prime} \odot Z_{1} , \end{align*}

(21) \begin{align*} & Z_{im2} = Z_{s}^{\prime} \odot Z_{2} , \end{align*}

where $\odot $ represents Hadamard product. $Z_{im1}$ and $Z_{im2}$ represent the embedding of specific graph after imputing. Then, we perform clustering by combining the representations from the specific graph and the shared graph:


(22) \begin{align*} Z_{final} = \theta Z_{im1} + (1 - \theta) Z_{im2}, \quad\text{s.t.} \quad 0 < \theta < 1.\end{align*}

### scAGCI algorithm

The algorithm of scAGCI is given in Algorithm 1.



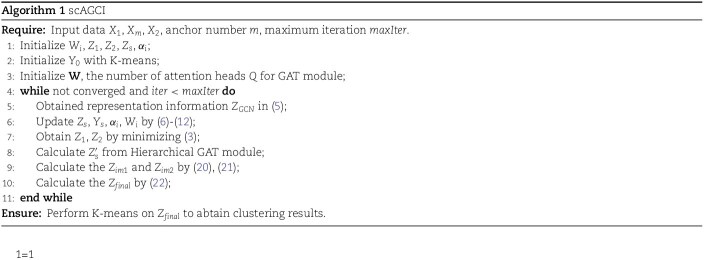



### Complexity analysis

Due to the application of anchor graph learning, scAGCI has a low computational complexity analysis. It mainly involves the iterative update data of four variables: the shared or specific graph representation $Z$, the anchor matrix $Y$, the weight coefficient $\alpha _{i}$, and anchor projection matrix $W_{i}$, derived from Singular Value Decomposition (SVD), graph learning, and matrix multiplication. It costs $O(n^{2}+nml+ml^{2}+m^{3})$, where $m<<n, l<<n$. The graph attention module costs $O(m^{2})$ and the generic fusion completion module, which mainly involves matrix multiplication and linear computation, costs $O(nm)$.

## Data and experiments design

### Datasets and preprocessing

In our study, we utilize five multi-omics datasets from different platforms, as shown in Table 1. For the CellMix Dataset [36], we use the Seurat package [9] to retain the top 500 highly expressed genes in the scRNA-seq dataset and the 7136 peaks within 100 kbp upstream and gene body in scATAC-seq. For the PBMC-3k Dataset (Human Peripheral Blood Mononuclear Cell) (https://support.10xgenomics.com/single-cell-multiome-atac-gex/datasets/1.0.0/pbmc_granulocyte_sorted_3k), we use Azimuth software [9] to predict 2900 cell types and delete cell type data with less than five cells. To improve efficiency, we apply the Seurat package [9], retaining the top 1500 highly expressed genes and the 11 063 peaks in the 100 kbp upstream and gene bodies in the scATAC-seq data. A SNARE-seq2-based dataset of 5081 cells from P0 mouse cortex (Mouse-P0), generated by droplet-based profiling, to test paired omics integration [9]. The Mouse Skin Dataset (Mouse-skin) [37] is preprocessed. The BMMC Dataset (Bone Marrow Mononuclear Cells) [38] is collected from 12 healthy human donors to support Multimodal Single-Cell Data Integration Challenge at NeurIPS 2021, 120 000 single cells from the human bone marrow with two commercially available multi-modal technologies.

**Table 1:** Summary of the datasets after preprocessing

Datasets	Cells	Genes	Peaks	Subtypes
Cellmix	1047	500	7136	4
PBMC-3k	2847	1500	11 063	14
Mouse-P0	5081	19 322	229 429	19
Mouse-skin	34 774	2000	35 943	23
BMMC	69 249	23 296	120 010	22

We input the preprocessed data into other comparison models separately. For our method, we input the two groups separately. The omics data are used as two omics views, and then the data after feature merging are inputted into the model together as a merging view.

### Comparison methods and evaluations metrics

Our method is compared with 13 other SOTA clustering methods, which mainly include classic methods such as K-means(features-merged dataset), methods based on matrix or factor decomposition, such as Liger [11], MOFA+ [7], scAI [8], UnionCom [12], and JSNMF [39], encoder-based methods DCCA [14], scMVAE [15], and scMVP [16], methods based on contrastive learning: scMCs [22], and anchor graph learning-based clustering: EOMSC-CA [32]. Except for anchor graph clustering, a multi-view method, the others are single-cell multi-omics clustering methods. The specific parameter settings follow the original study.

We evaluate the performance of the clustering result using accuracy (ACC), normalized mutual information (NMI), F1-score, precision, recall [40], adjusted rand index (ARI), and Silhouette Coefficient(S-score) [41]. The ACC, NMI, F-score, precision, Recall, and ARI range are all [0,1]. The S-score is [−1,1]. Some require ground truth labels, and we use the cell-type labels provided on the original paper. These evaluations are carried out on five datasets of different sizes and are compared visually with our method. These indicators comprehensively consider the accuracy, stability, and consistency of clustering results.

### Experiments design

We initialize the GCN with two layers. The initial learning rate is 0.01; iterations are 1000 times; the weight decays weight decay set 5e-3, and the Adam optimizer is used for learning. Besides, the number of anchors needs to be determined within the range $[10k, 20k, 30k, 40k, 50k, 60k]$, where $k$ denotes the number of clusters in the dataset. For GAT, we set the number of GAT layers to 2 and the multi-head attention to 3. The completion operator is set to 0.5:0.5. We conduct 10 experiments using random seeds to ensure randomness, obtaining average results for ACC, NMI, F-score, precision, recall, ARI, and S-score while validating the results every 30 iterations.

As for the comparison methods, we adopt the parameters that yielded the best results in the original paper. scAI, EOMSC-CA, and JSNMF are implemented on MATLAB. Both MOFA+ and Liger are implemented on Rstudio(version 2021). All algorithms are run on a machine with Intel(R) Xeon(R) Gold 6348 CPU @ 2.60GHz addressing 100GB memory and one NVIDIA A30 TENSOR CORE GPU.

## Performance evaluation

### Evaluate algorithm performance in comparison with SOTA methods

To maintain the clarity and conciseness of the main manuscript, we have included the results from the Mouse-P0 dataset in the supplementary materials. Table 2 shows the clustering results of our method and 13 other methods on other four datasets. The values marked in bold are the best results among all methods, and the values underlined are sub-optimal. We conduct the following findings.

**Table 2:** Comparing results under different methods

Dataset	Methods	ACC	NMI	F1-score	Precision	Recall	ARI	S-score
Cellmix	K-means	0.6705	0.5950	0.6300	0.6351	0.6249	0.4563	0.5932
	Liger	0.8758	0.8338	0.8767	0.8900	0.8637	0.8637	0.6921
	MOFA+	0.8959	0.8529	0.8960	0.8897	0.9023	0.8978	0.6907
	scAI	0.6523	0.6122	0.6830	0.6415	0.7303	0.5450	0.6814
	UnionCom	0.8510	0.7058	0.8815	0.8836	0.8795	0.6420	0.5236
	DCCA	0.6561	0.6189	0.6502	0.6491	0.6513	0.5132	0.6395
	scMVAE-POE	0.9427	0.8538	0.9344	0.9237	0.9453	0.8397	0.6788
	scMVAE-NN	0.9389	0.8176	0.8804	0.8667	0.8946	0.8093	0.6532
	scMVAE-Direct	0.9083	0.8109	0.8889	0.8746	0.9037	0.8115	0.6854
	JSNMF	0.5263	0.2617	0.2295	0.5326	0.1463	0.1965	0.3015
	scMVP	0.8691	0.8567	0.8711	0.8920	0.8514	0.8756	0.7032
	EOMSC-CA	0.7832	0.7851	0.8420	0.8525	0.8318	0.7223	0.7132
	scMCs	0.9436	0.9073	0.9389	0.9330	0.9448	0.9391	0.6664
	scAGCI	**0.9570**	**0.9475**	**0.9572**	**0.9562**	**0.9583**	**0.9522**	**0.7263**
PBMC-3k	K-means	0.4555	0.5678	0.3752	0.3837	0.3671	0.4835	0.4952
	Liger	0.5397	0.5745	0.4271	0.4632	0.3962	0.4369	0.3691
	MOFA+	0.4596	0.4189	0.4583	0.3916	0.5523	0.5735	0.5245
	scAI	0.5609	0.6468	0.3960	0.4847	0.3347	0.5932	-0.0444
	UnionCom	0.5094	0.6065	0.5295	0.5262	0.5328	0.4001	0.4698
	DCCA	0.3911	0.5943	0.3616	0.3549	0.3686	0.4069	0.3865
	scMVAE-POE	0.5640	0.6037	0.5220	0.5195	0.5246	0.4563	0.4987
	scMVAE-NN	0.5717	0.6113	0.6105	0.6113	0.6097	0.6373	0.5586
	scMVAE-Direct	0.5317	0.5052	0.5853	0.5936	0.5773	0.4442	0.2198
	JSNMF	0.4614	0.4162	0.3073	0.2947	0.3211	0.2846	0.2456
	scMVP	0.3528	0.5863	0.4489	0.4296	0.4700	0.6451	0.4625
	EOMSC-CA	0.4412	0.4475	0.4720	0.3745	**0.6383**	0.3465	0.4936
	scMCs	0.3855	0.5348	0.3903	0.3604	0.4257	0.5960	0.5292
	scAGCI	**0.6294**	**0.6962**	**0.6364**	**0.6514**	0.6220	**0.6579**	**0.5631**
Mouse-skin	K-means	0.4344	0.4652	0.4515	0.5144	0.4835	0.2368	0.1037
	Liger	0.3047	0.2846	0.2481	0.2500	0.2463	0.2333	0.1078
	MOFA+	0.4839	0.5149	0.4984	0.5031	0.4939	0.3026	0.1095
	scAI	0.5217	0.4937	0.5076	0.4989	0.5167	0.3437	0.0081
	UnionCom	NA	NA	NA	NA	NA	NA	NA
	DCCA	0.2887	0.2650	0.3241	0.2936	0.3619	0.2593	-0.0004
	scMVAE-POE	0.5463	0.5170	0.5916	0.5776	0.6063	0.3300	0.0752
	scMVAE-NN	0.5037	0.4932	0.5503	0.5459	0.5548	0.2950	0.1249
	scMVAE-Direct	0.5412	0.5240	0.5801	0.5479	0.6164	0.3310	0.0794
	JSNMF	0.1703	0.1401	0.1410	0.1425	0.1396	0.0872	0.2537
	scMVP	0.3346	0.2936	0.3183	0.3223	0.3145	0.3063	0.3033
	EOMSC-CA	0.3843	0.3063	0.3975	0.4539	0.3536	0.1986	0.3023
	scMCs	0.3428	0.4332	0.2440	0.2140	0.2838	0.2608	0.4088
	scAGCI	**0.5631**	**0.5259**	**0.6134**	**0.5978**	**0.6300**	**0.3567**	**0.4360**
BMMC	K-means	0.3843	0.4831	0.4197	0.3978	0.4442	0.3034	0.1834
	Liger	0.4362	0.5233	0.4187	0.4212	0.4162	0.2889	0.1563
	MOFA+	0.4963	0.6065	0.4481	0.4529	0.4434	0.2724	0.1680
	scAI	NA	NA	NA	NA	NA	NA	NA
	UnionCom	NA	NA	NA	NA	NA	NA	NA
	DCCA	0.3655	0.4937	0.3634	0.3779	0.3501	0.3923	0.1891
	scMVAE-POE	0.4888	0.6250	0.4634	0.4739	0.4534	0.4160	0.1969
	scMVAE-NN	0.5204	0.5870	0.4658	0.4775	0.4548	0.4041	0.2866
	scMVAE-Direct	0.5512	0.6740	0.5582	0.5708	0.5463	0.4220	0.2309
	JSNMF	NA	NA	NA	NA	NA	NA	NA
	scMVP	0.5314	0.6137	0.4704	0.4936	0.4493	0.3964	0.2596
	EOMSC-CA	NA	NA	NA	NA	NA	NA	NA
	scMCs	0.5190	0.6196	0.4812	0.5089	0.5269	0.4080	0.2236
	scAGCI	**0.5790**	**0.6755**	**0.5745**	**0.5799**	**0.5693**	**0.4880**	**0.3069**

We observe that scAGCI always achieves satisfactory embeddings compared with other methods. For small to medium-sized datasets, compared with the suboptimal method scMCs, our method has largely improved, with indicators improved by 1.34%, 4.02%, 1.83%, 2.32%, 1.35%, 1.31%, and 5.99% on ACC, NMI, F1-score, Precision, Recall, ARI, and S-score on the Cellmix dataset. On the PBMC-3k dataset, our method improved the index by 5.77%, 8.49%, 2.59%, 4.01%, −1.23%, 2.42%, and 0.45% compared with the scMVAE-NN. On the Mouse-P0 dataset (see Table S1 in the Supplementary), our method improved the index by 3.53%, 7.43%, 4.22%, 4.58%, 3.88%, 3.52%, and 20.51% compared with the suboptimal method.

For large-sized datasets, our method also has an improvement effect visible. Compared with the suboptimal method scMVAE-POE, our method has improved by 1.68%, 0.89%, 2.18%, 2.02%, 2.37%, 2.67%, and 36.72%, respectively, on Mouse-skin dataset. Especially on the larger sized dataset with >100 000 single cells, our method has also achieved an improvement of 2.78%, 0.15%, 1.63%, 0.91%, 2.30%, 6.60%, and 2.03%, respectively, compared with the suboptimal method scMVAE.

Its outstanding performance mainly benefits from the extraction of specific omics information, the mining of high-order shared information, and the full fusion of these information. Regarding the other comparison methods, scMCs takes into account shared and specific information extraction, but it misses the high-order shared information. Other methods pay more attention to the extraction and completion of specific information from omics.

We further find that scAGCI significantly alleviates sparsity. For instance, on the CellMix dataset, the matrix decomposition-based methods scAI and Unioncom have ARIs of 0.5450 and 0.6420, respectively. Methods using ZINB decoders to reconstruct data like scMVAE and scMCs achieve ARI values above 0.8, while we attain an ARI of 0.9522. The lower ARI indicates that they performed poorly in addressing the sparsity of single-cell datasets. scAGCI retains better sparsity alleviation effects through the multi-view subspace anchor co-optimization method, further improving the clustering discriminability of the integrated embeddings. The results are also notable in the PBMC-3k, Mouse-skin, and BMMC datasets.

### Ablation study

scAGCI consists of three main modules: the Multi-view Subspace Anchor Co-optimization Module (MAC), the Hierarchical GAT Module (H-GAT), and the Commonality Fusion Completion Module (CFC). The MAC extracts specific and shared information from omics data, the H-GAT explores high-oder shared information, and the CFC complements specific information and integrates shared and specific information. The three modules contribute to the excellent performance of scAGCI in integrating omics information. We conduct ablation experiments on four datasets to demonstrate the effectiveness of each module in our method as shown in Table 3.

**Table 3:** Ablation study

Dataset	MAC	H-GAT	CFC	ACC	ARI	NMI
CellMix				0.7832	0.7223	0.7852
	✓			0.9465	0.8346	0.8493
	✓	✓		0.9501	0.8793	0.8646
	✓	✓	✓	0.9570	0.9522	0.9475
PBMC-3k				0.4412	0.3465	0.4475
	✓			0.5894	0.5579	0.6454
	✓	✓		0.6002	0.5724	0.6531
	✓	✓	✓	0.6294	0.6579	0.6962
Mouse-skin				0.3827	0.1988	0.2933
	✓			0.4936	0.3027	0.4978
	✓	✓		0.5414	0.3374	0.5063
	✓	✓	✓	0.5631	0.3567	0.5259
BMMC				0.3612	0.2899	0.4036
	✓			0.5364	0.4187	0.5833
	✓	✓		0.5563	0.4793	0.6463
	✓	✓	✓	0.5790	0.4880	0.6755

Due to MAC module, our results are improved by 16.33%, 11.23%, and 6.4% on CellMix, 14.82%, 21.14%, and 19.79% on PBMC-3k, 11.09%, 10.39%, and 20.45% on Mouse-skin, and 17.52%, 12.88%, and 17.97% on BMMC. The results show that the specific graph representation, shared graph representation, and anchor co-optimization trained by GCN are beneficial in enhancing model performance. This is mainly due to aggregating feature information such as genes and peaks into nodes to obtain more representative anchors.

On this basis, we add the H-GAT module. We observe that the clustering effect is improved by 0.36%, 4.47%, and 1.53% on Cellmix, 1.08%, 1.49%, and 0.77% on PBMC-3k, 4.78%, 3.47%, and 0.85% on Mouse-skin, and 1.99%, 6.06%, and 6.30% on BMMC. The module explores the high-order information shared between the peak and gene, adaptively adjusting weights for different neighbors, thus improving the model effect.

Finally, we add the CFC module. Our results improve by an average of 2.01%, 4.66%, and 4.38% on four datasets. This illustrates that using high-order shared information to complete specific information and then fuse it helps to obtain a more differentiated cell representation while retaining the shared information of the cell.

The above ablation results demonstrate the effectiveness of each module. They also show that the complete model using all of them has better clustering results.

### The effects of parameters on the clustering performance

**Figure 2:**
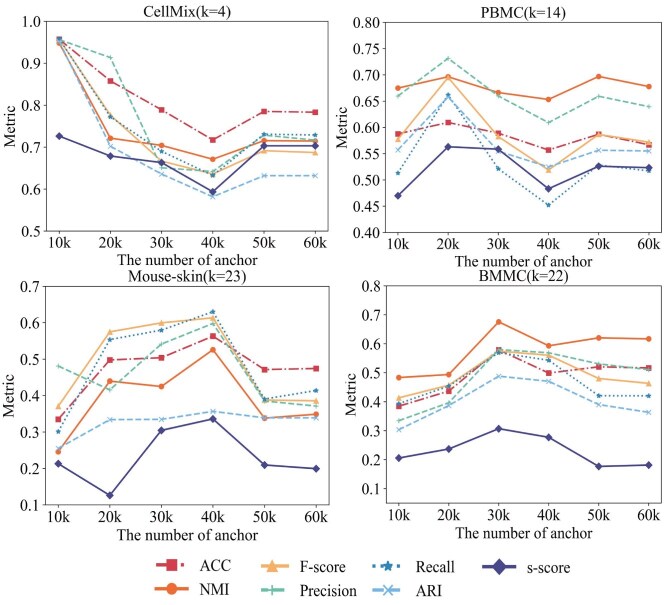
Effects of parmeter $m$ on the seven evaluation measures under different datasets ($k$ denotes the number of cluster of the dataset).

To analyze the impact of the number of anchors on performance, we conducted experiments on four datasets, as depicted in Fig. 2. We varied the number of anchors within the range of 10k to 60k (with a step size of 10k) and implemented our method on different datasets.

For the Cellmix dataset, we recommend setting the anchor point count at 10k, which allows for effective capture of key cell type features while minimizing computational overhead, given its relatively small size. For the PBMC-3k dataset, we suggest setting the anchor point count at 20k. This will enhance clustering accuracy by increasing the ability to capture the heterogeneity and subtle distinctions between various immune cell types. In the Mouse-skin dataset, we set the anchor point count at 40k, which yielded optimal results. This is due to the presence of many cells with similar gene expression profiles, enabling better differentiation among closely related cell types. For the BMMC dataset, we recommend selecting 30k anchor points.

### scAGCI can distinguish cells compared with single omic

**Figure 3:**
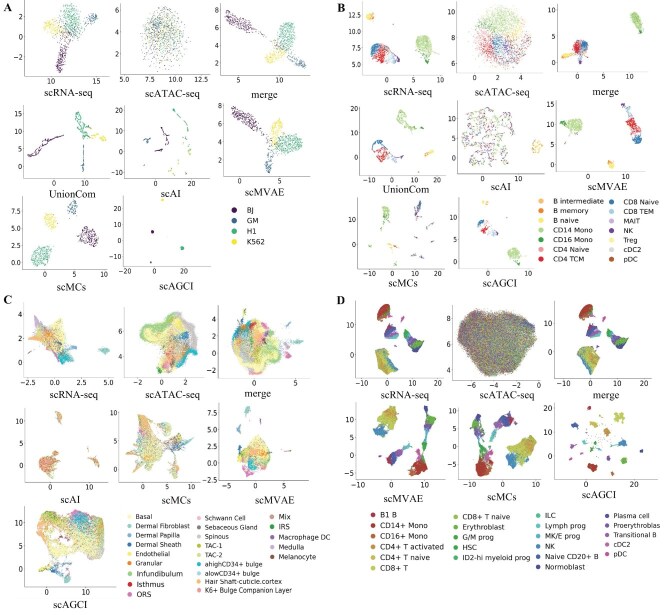
UMAP scatterplots for CellMix, PBMC-3k, Mouse-skin, and BMMC are shown in subfigures A, B, C, and D, with cells colored by their true cell type.

To demonstrate the advantages of multi-omics in identifying cell types, Fig. 3 compares single-omics and integrated analyses across four datasets (A: CellMix, B: PBMC-3k, C: Mouse-skin, D: BMMC). Each subfigure includes visualizations of raw scRNA-seq, scATAC-seq, feature-merged data, results from other methods (UnionCom, scAI, scMVAE, scMCs), and our method (scAGCI). Methods that failed to run are not visualized. Moreover, the additional UMAP plot of Mouse-P0 displays in the Supplementary Figure S2.

On the CellMix dataset, raw scRNA-seq data could clearly cluster most cells into four cell clusters, but some mixing are observed in the BJ, GM, and H1 cells. The raw scATAC-seq data show a higher clustering degree for the K562 cell than others in Fig. 3A scRNA-seq, scATAC-seq. On the PBMC-3k dataset, Mouse-skin, BMMC, and Mouse-P0 dataset, although the clustering boundaries are unclear, the clustering effect of raw scRNA-seq can effectively group similar cells together, shown in Fig. 3B–D and Supplementary Figure S2. However, in scATAC-seq, direct clustering cannot distinguish cell types, and scATAC-seq only shows clear distinctiveness in some cell types.

### scAGCI further distinguishes cell subtypes

To exhibit the unique advantages of scAGCI in cell type clustering, we visualize UnionCom, scAI, scMVAE, scMCs, scAGCI, and features-merging using UMAP visualization in Fig. 3.

Among the visualized integration methods on the CellMix in Fig. 3A, scAI clearly distinguishes between GM and BJ cells, but H1 and K562 cells are mixed. UnionCom, scMVAE, and scMCs differentiate the four cell types with distant inter-class distances. In contrast, our method, benefiting from the multi-view space anchor co-optimization module, tightly clusters each cell type with smaller inter-class distances.

As shown in Fig. 3B, all visualized integration methods separate B cells from other immune cells on the PBMC-3k dataset. Methods like scMVAE or UnionCom demonstrate significant inter-class distances among B cells, CD14 Mono, CD16 Mono, cDC, and various T cells. Yet, they struggle to cluster specific subtypes accurately, such as CD4 Naive, CD4 TCM, CD8 Naive, and CD8 TCM. Our method, however, clearly delineates these subtypes, showcasing its superior capability. Similarity, on the Mouse-P0 dataset (Figure S2 in the Supplementary), scAGCI successfully differentiates between Peri, CR, and other cell types.

We visualize these methods on the mouse-skin dataset as well in Fig. 3C. Unfortunately, the UnionCom method fails to run due to the dataset’s large size. Compared with scAI, other methods are able to cluster cell types such as Dermal Fibroblast, Dermal Papilla, Dermal Sheath, Macrophage DC, and Melanocyte. Ours also clusters the Schwann Cell cluster and the Hair Shaft-cuticle cluster cortex cell cluster.

In Fig. 3D, scAGCI successfully differentiates between CD14+ mono and CD16+ mono, as well as activated CD4+ T cells and naive CD4+ T cells, further highlighting its effectiveness in large-scale data analysis on the BMMC dataset.

### scAGCI retains the specific information of scRNA-seq

**Figure 4:**
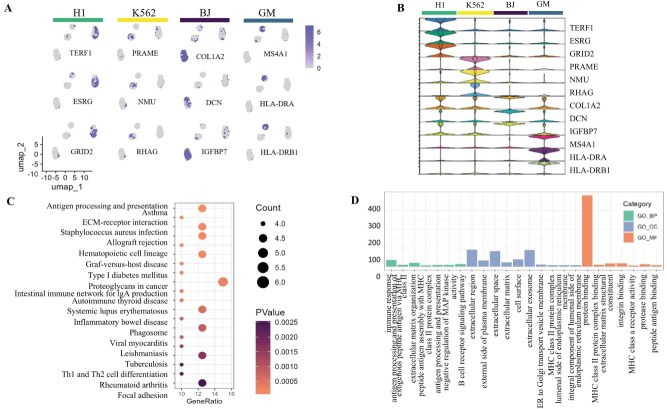
Analysis results of Cellmix scRNA-seq dataset (A: UMAP plots of Top 3 marker genes for per cell type; B: Violin plots of 12 marker genes from A; C: KEGG pathway enrichment; D: GO term enrichment).

scRNA-seq captures transcriptional profiles to reveal gene expression patterns. We take the human datasets (CellMix and PBMC-3k) as examples to verify the specific information effectiveness of the scRNA-seq and scATAC-seq extracted by our method. We conduct differential gene expression analysis and enrichment analysis on the specific information extracted from the scRNA-seq data on the CellMix and PBMC-3k datasets. In this study, we use the Seurat package to identify the top three genes with the greatest differences between different cell clusters as candidate genes. Then, we display the distribution of these differentially expressed genes in the cell clusters in Fig. 4A, B. According to known information [42, 43], the CellMix dataset consists of human embryonic stem cells (H1), B lymphocytes (GM12878), blood cancer lymphocytes (K562), and human skin fibroblasts (BJ). The PRAME, COL1A, and HLA-DRB1 are considered known marker genes for K562, BJ, and GM, respectively. According to the CellMarker [44] database, TERF1, ESRG, and GRID2 are marker genes for embryonic tissue. Figure 4A shows the expression of these genes in H1 cells, which is consistent with the actual differential gene expression in cells.

In addition, we perform KEGG enrichment analyses and gene ontology (GO) term enrichment on genes. In KEGG pathway analysis in Fig. 4C, we notice the enrichment in antigen processing and presentation and focal adhesion. The GO enrichment analysis on Cellmix data reveals several findings in Fig. 4D. We observe enrichment in molecular functions associated with protein binding, including MHC class II proteins and integrins. Biological processes such as antigen processing and presentation, antigen receptor activity are enriched. In terms of cellular components, we observe enrichment in extracellular exosomes and endoplasmic reticulum membranes.

**Figure 5:**
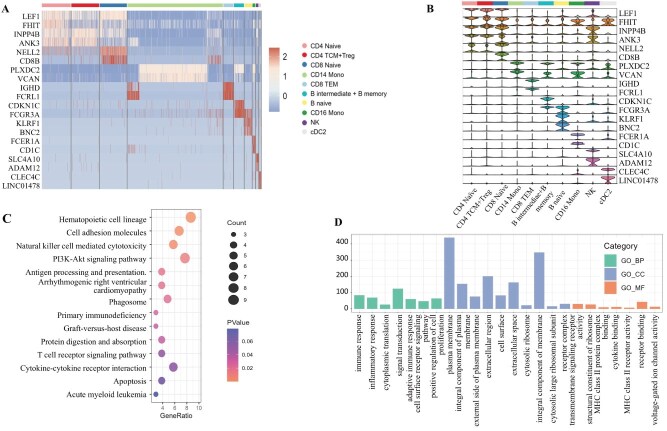
Analysis results of the PBMC-3k scRNA-seq dataset (A: Heatmap; B: Violin plots across cell types; C: KEGG pathway enrichment; D: GO term enrichment).

Similarly, in the specific information extracted from scRNA-seq on the PBMC-3k dataset, we select the top two genes with the greatest differences between each cell cluster and other cells as candidate genes in Fig. 5A and B. The PBMC-3k dataset consists of single-cell from human peripheral blood, mainly including lymphocytes (T cells, B cells, and NK cells), monocytes, dendritic cells, etc. The omics-specific information of scRNA-seq blurs the boundaries among CD4 TCM and Treg cells, B intermediate cells, and B memory cells, which is also reflected in previous single-omics visualizations.

Figure 5C shows primary immunodeficiency and T cell receptor signaling pathways may correspond with the immune response, inflammatory response in KEGG pathway enrichment. Besides, Hematopoietic cell lineage, Cell adhesion molecules, and Antigen processing and presentation were enriched in KEGG. In Fig. 5D, we observe cellular components term enriched in plasma membrane, integral components of membranes, and receptor complexes. In biological processes, significant enrichments are detected in signal transduction and cell surface receptor signaling. Additionally, immune response, inflammatory response, and cytoplasmic translation are also observed to be enriched. Regarding molecular functions, we view enrichments in protein binding and structural constituents of ribosomes. These findings collectively suggest that genes within this dataset play pivotal roles in cellular membrane and receptor-related activities and immune responses.

### scAGCI retains the specific information of scATAC-seq

**Figure 6:**
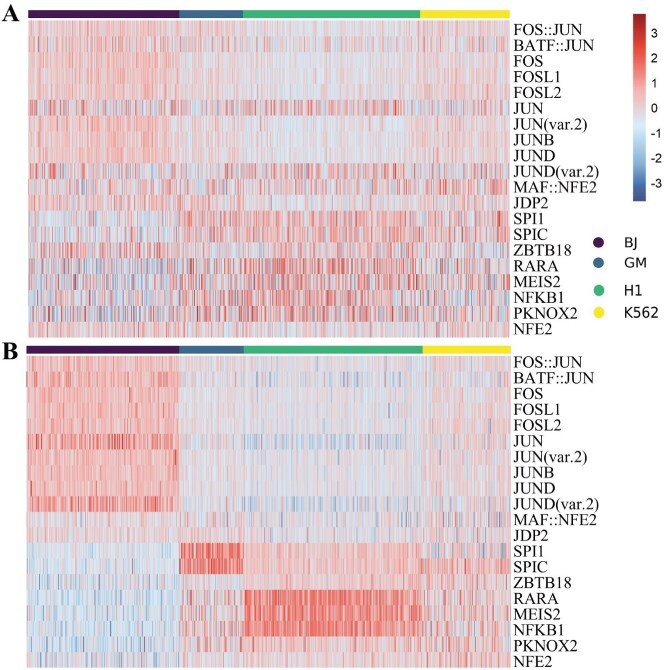
Heatmap of TF activity scores using ChromVAR by cell type for the CellMix scATAC-seq dataset: raw data (Panel A) and scAGCI-processed features (Panel B).

scATAC-seq elucidates chromatin region accessibility, reflecting regulatory elements controlling gene expression. To demonstrate our method’s capability in preserving specific biological information, we calculate the transcription factor (TF) activity scores by the popular tool, chromVAR (v1.6.0) [45] for both the raw and extracted scATAC-seq specific omics data in Fig. 6A and B. The TF activity score quantifies the regulatory potential of TF in a given cell type, derived from scATAC-seq chromatin accessibility profiles.

**Figure 7:**
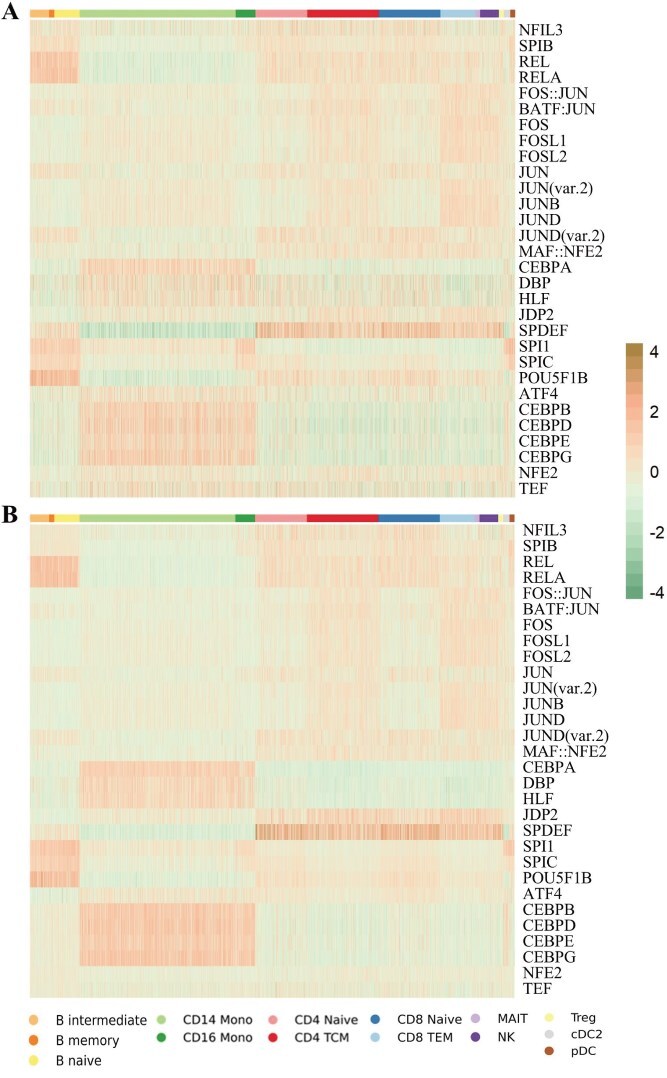
Heatmap of TF activity scores using ChromVAR by cell type for the PBMC-3k scATAC-seq dataset: raw data (Panel A) and scAGCI-processed features (Panel B).

As can be seen in Fig. 6, the extracted specificities are as follows: JUN, JUND, FOS, BATF::JUN in BJ cells, NFK1, RARA in H1 cells, SPl1 in GM12878 cells, and FOS, JUND co-associate in K562 but not in GM12878 [37, 44]. Figure 7A and B visualizes the scATAC-seq data from PBMC-3k. B cells have higher activity scores in REL, RELA, and POU5F1B TFs, while the activity level of memory cells in SPDEF TFs is much lower than that of other kinds of cells. The expression of CD16 Mono on SPl1 and SPlB is slightly higher than that of CD14 Mono. Finally, in many T cells, the TF SPDEF is highly expressed, and Naive cells have lower scores on FOS and JUN than TCM cells [46].

The difference in results between the CellMix and PBMC-3k datasets (as shown in Fig. 6 and Fig. 7) can be attributed to the characteristics of the datasets and the role of preprocessing. In the case of PBMC-3k, the raw data for both omics profiles of different cell types are already well-separated (as shown in the top row of Fig. 3B), meaning that preprocessing has limited potential for further improvement. In contrast, the CellMix dataset shows that the raw data of the scATAC-seq profile are mixed together (top row of Fig. 3A), and preprocessing with scAGCI effectively separates the integrated data (bottom row of Fig. 3A). Therefore, scAGCI provides significant improvements for CellMix, where preprocessing helps to better differentiate the cell types, but the same level of improvement is not observed in the PBMC-3k dataset due to its already well-separated raw data.

### scAGCI saves time cost

Considering the increasing scale of single-cell sequencing datasets, the runtime on analysis is a critical concern. We record the time consumption of each method on datasets of varying sizes.

As depicted in Table 4, on the CellMix dataset, UnionCom has the shortest runtime, followed by our method, while scAI consumes the most time. Our method demonstrates the shortest runtime, followed by the UnionCom algorithm on the PBMC-3k dataset. On the Mouse-skin dataset, UnionCom fails to run on large-scale datasets, while our algorithm not only runs but also has the shortest runtime. In small datasets (Cellmix), the runtime of UnionCom based on matrix decomposition is more advantageous, but our method performs better in the clustering effect. For medium(PBMC-3k) and large datasets (Mouse-skin, BMMC datasets), our method has an obvious advantage in terms of runtime and clustering effect.

**Table 4:** Different scale datasets runtime (minute)

	CellMix	PBMC-3k	Mouse-skin	BMMC
Unioncom	0.98	5.55	NA	NA
scAI	25.47	30.59	635.18	NA
scMVAE	2.76	5.43	162.65	200.36
scMCs	3.60	6.86	181.65	256.69
scAGCI	1.01	2.76	150.924	183.66

## Conclusion

In this work, we propose scAGCI, an anchor graph-based method for cell clustering from scRNA-seq and scATAC-seq, to address the challenge of heterogeneity and high sparsity. We take the structure and feature information from each omics and shared anchor graph during the dynamic anchor learning strategy. We mine the high-order shared information to rich the relationship between peak and gene. Experimental results demonstrate that scAGCI exhibits excellent capabilities in distinguishing different cell types and subtypes. scAGCI addresses the limitations of anchor graphs in representing omics data by co-optimizing anchor graph and graph embedding methods, resulting in reduced integration time for large-scale datasets and decreasing sparsity and noise in omics data.

Multi-omics integration study helps uncover various aspects of cell function, expression dynamics, and metabolic status, leading to a deeper understanding of cell diversity and complexity. It aids in exploring disease-specific omics features and molecular mechanisms, offering insights into disease causes and mechanisms. This framework can be extended to other omics modalities (e.g. proteomics) and enhanced through automated anchor selection algorithms. Integration with large language models [47, 48] will further improve biological interpretation, advancing multi-omics analysis capabilities.

**Key Points**

- We take three types of single cell multi-omics’ information into account. The first one is structure information, i.e. cell-to-cell connections, which the previous works often take. The second one is feature information of each omic, for instance, gene feature in scRNA-seq data and peak feature in scATAC-seq data. The last one is their shared high-order information, like the relationship between gene and peak, which is seldom in the existing studies, but is significant for integrating single-cell multi-omics. Compared with only one piece of information, this combination information more fully represents single-cell multi-omics. They are applied to construct each omic’s specific graph and their shared high-order graph.
- To optimize the specific and shared representation of single-cell multi-omics, we design a dynamic anchor learning strategy. We implemented GCNs to optimize each and shared omic’s anchors and their anchor graphs by a united and bi-directional cycle. It effectively reduces the sparsity of omic data.
- We develop a hierarchical GAT to further mine the higher order information of shared representation, which helps us distinguish different cell subtypes. Furthermore, we carry out a series of comparison experiments. The results illustrate our method’s outstanding performance on single-cell multi-omics clustering tasks. It fully preserves the raw single-cell omic data, which reflects regulatory elements controlling gene expression.

## Supplementary Material

Supplymentary_bbaf244

## Data Availability

The codes are available at https://github.com/hebutdy/scAGCI.
