# Supplementary material for: scAGCI: an anchor graph-based method for cell clustering from integrated scRNA-seq and scATAC-seq data
Source: Brief Bioinform. 2025 Jul 7;26(4):bbaf244. doi: 10.1093/bib/bbaf244 (PMC12232420; doi:10.1093/bib/bbaf244)
Supplement: Supplymentary_bbaf244 [file supplymentary_bbaf244.pdf]

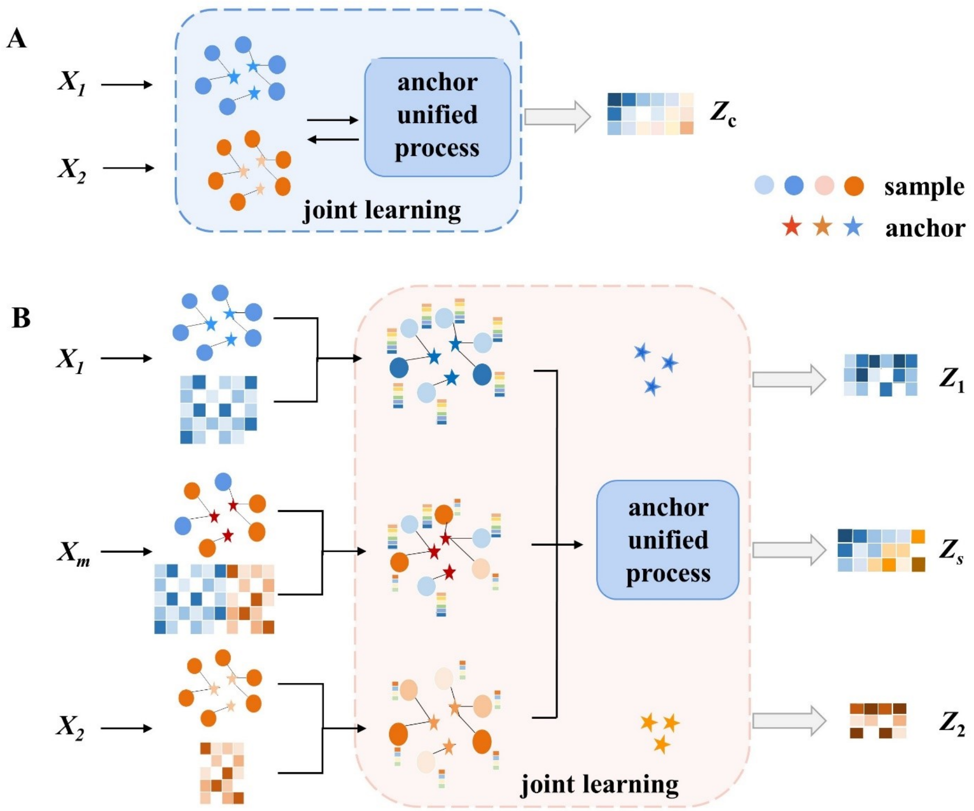

Fig. S1. The difference on anchor graph learning mechanism between the EOMSC-CA model(Panel A) and our model (Panel B). (i) The EOMSC-CA model only applies the structure information of graph to cluster. In contrast, we aggregate feature information of neighbors by three GCNs. (ii) By jointing learning of the anchor graph and anchor, the EOMSC-CA model learns consensus anchor Z_c during the anchor unified process, while we capture not only shared representation Z_s from the anchor unified process but specific information of each view Z_1 , Z_2 . X_1, X_2 and their merging data X_m are input.

Table S1. Comparing results on Mouse-P0 dataset

Methods	ACC	NMI	F1	Precision	Recall	ARI	SC
K-means	0.1584	0.1289	0.1502	0.1421	0.1629	0.1593	0.0406
Liger	0.3054	0.2243	0.3240	0.3314	0.3170	0.2713	0.1073
MOFA+	0.4681	0.3989	0.3991	0.4011	0.3972	0.2831	0.1022
scAI	0.0801	0.0122	0.0676	0.0754	0.0613	0.0408	-0.0023
UnionCom	0.2771	0.2001	0.2663	0.2652	0.2676	0.2212	0.1030
DCCA	0.3047	0.3349	0.2916	0.2749	0.3106	0.2843	0.1622
scMVAE-POE	0.2708	0.2540	0.2604	0.2697	0.2518	0.1330	0.0805
scMVAE-NN	0.3840	0.3430	0.3529	0.3864	0.3249	0.2080	0.1246
scMVAE-Direct	0.4542	0.3901	0.4406	0.4463	0.4351	0.3780	0.0552
JSNMF	0.2049	0.1345	0.1418	0.1531	0.1321	0.1818	0.0724
scMVP	0.3636	0.3054	0.4096	0.4227	0.4165	0.4075	0.1436
EOMSC-CA	0.3694	0.3146	0.3357	0.3602	0.3144	0.2631	0.1676
scMCs	0.2777	0.3246	0.2740	0.2523	0.2998	0.2656	0.1473
scAGCI	**0.5034**	**0.4732**	**0.4828**	**0.4921**	**0.4739**	**0.4427**	**0.3727**

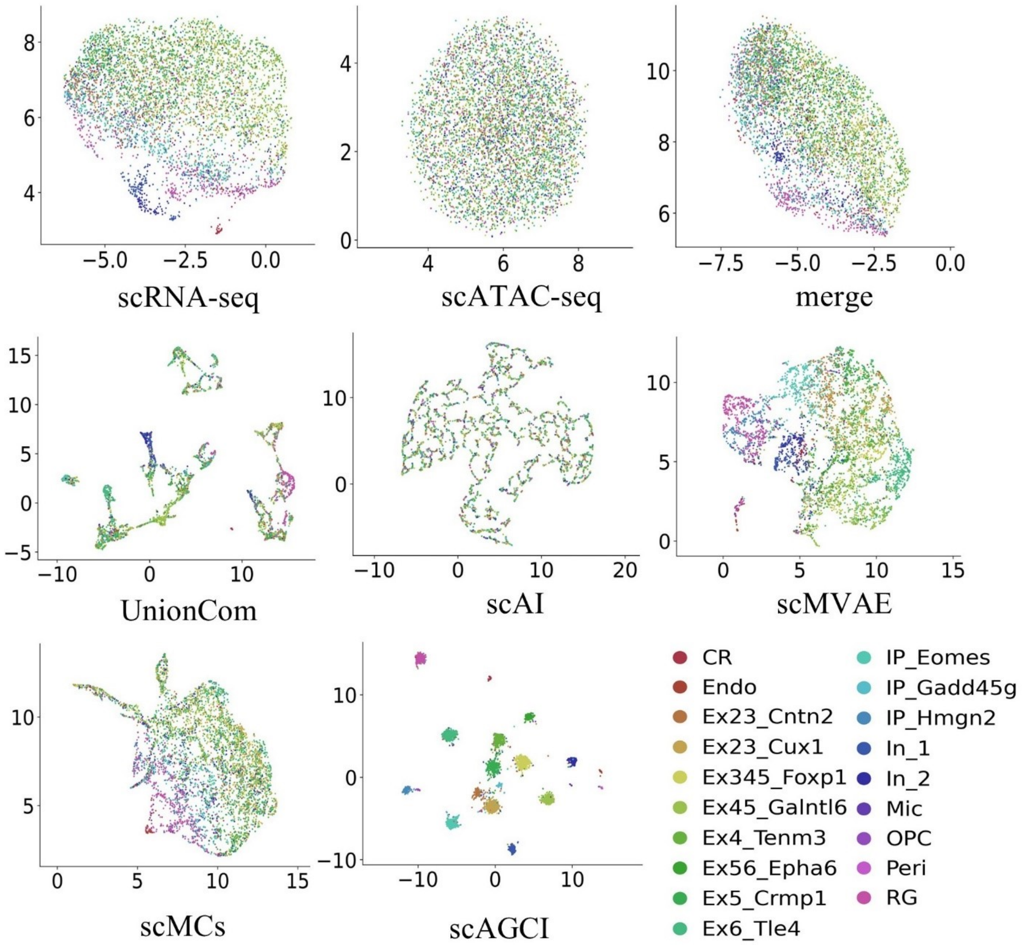

Fig. S2. UMAP scatterplots for Mouse-P0 with cells colored by their true cell type. It includes visualizations of raw scRNA-seq, scATAC-seq, feature-merged data, results from other methods (UnionCom, scAI, scMVAE, scMCs), and our method (scAGCI).
